# Advancements in the investigation of radioactive microspheres for brachytherapy

**DOI:** 10.3389/fbioe.2025.1621418

**Published:** 2025-07-16

**Authors:** Xiaohui Jiang, Lei Chen, Xiao Xu

**Affiliations:** 1 College of Physics and Electronic Engineering, Jining University, Jining, China; 2 Cancer Center, Dongguan Engineering Research Center for Innovative Boron Drugs and Novel Radioimmune Drugs, The 10th Affiliated Hospital of Southern Medical University, Southern Medical University, Guangzhou, China; 3 Guangdong Engineering Research Center of Boron Neutron Therapy and Application in Malignant Tumors, The 10th Affiliated Hospital of Southern Medical University, Southern Medical University, Guangzhou, China

**Keywords:** tumor, radiation, brachytherapy, radioactive microspheres, theranostics

## Abstract

In clinical practice, the management of most non-surgically resectable solid tumors necessitates a multidisciplinary treatment approach. Optimal solutions involve the integration of local and systemic treatments, such as targeted immunotherapy and chemotherapy. Micron-sized radioactive microspheres or particles have gained widespread application in the localized treatment of various organ tumors, encompassing liver cancer, lung cancer, tongue cancer, pancreatic cancer, head and neck cancer, ovarian cancer, bone cancer, among others. As such, the design and development of novel multifunctional radioactive microspheres constitute a crucial foundation for achieving effective local treatment in liver cancer and other cancer types. This article critically reviews the current developmental landscape, identifies challenges, and explores opportunities in the field of radioactive internal irradiation microspheres in recent years. The insights provided serve as a valuable reference for selecting and determining the developmental direction of clinical brachytherapy treatment carriers.

## 1 Introduction

Radioactive microspheres have emerged as a groundbreaking platform for locoregional cancer therapy, particularly in the treatment of unresectable solid tumors (Wu et al., 2023; Yang et al., 2023; Sun et al., 2025). By delivering high-dose radiation directly to tumor vasculature via intra-arterial administration, these microspheres enable precise tumor ablation while sparing surrounding healthy tissues—a principle central to brachytherapy (Filippi et al., 2020; Dong et al., 2024). Over the past decades, advancements in material science, radiochemistry, and interventional oncology have propelled radioactive microspheres into clinical practice, offering a minimally invasive alternative to conventional radiotherapy and systemic therapies for hepatocellular carcinoma (HCC), metastatic liver tumors, and other malignancies (Sun et al., 2025; Gupta et al., 2024).

The therapeutic efficacy of radioactive microspheres hinges on two critical components: the microsphere carrier and the encapsulated radionuclide (Wang L. et al., 2025; Pang et al., 2025). An ideal microsphere must exhibit 1) mechanical robustness to withstand vascular transport (Arranja et al., 2018); 2) chemical stability to prevent radionuclide leaching (Gupta et al., 2024); 3) biocompatibility to minimize systemic toxicity (Kühnel et al., 2022); and 4) tunable degradation kinetics for repeated treatments if required (Drescher et al., 2020a). Among radionuclides, β-emitters such as yttrium-90 (^90^Y) and holmium-166 (^166^Ho) dominate clinical use due to their moderate tissue penetration (8–12 mm) and favorable half-lives (Drescher et al., 2020b; Westcott et al., 2016; Morsink et al., 2024). Notably, ^166^Ho and lutetium-177 (^177^Lu) also emit γ-rays, permitting single-photon emission computed tomography (SPECT) imaging for real-time dosimetry—a feature absent in pure β-emitters like ^90^Y, which relies on suboptimal bremsstrahlung imaging (Cunha et al., 2023; Xiao et al., 2023).

Despite their promise, clinical translation faces persistent challenges. First, radionuclide leakage—observed in early-generation resin-based ^90^Y microspheres—can lead to off-target radiation exposure (Jiang et al., 2024). Second, imaging limitations complicate post-treatment verification; for instance, ^90^Y’s bremsstrahlung emissions produce low-resolution SPECT images (Debebe et al., 2019; Zade et al., 2013). Third, dose heterogeneity arises from uneven microsphere distribution in tumor vasculature, necessitating improved predictive dosimetry models (Wu X. et al., 2022). Recent innovations aim to address these issues: 1) Material engineering: Glass microspheres sintered with ^90^Y_2_O_3_ exhibit superior ^90^Y retention compared to resin counterparts (Gallio et al., 2016; Filippi et al., 2018). 2) Multimodal microspheres: Designs incorporating photothermal agents (e.g., ^131^I-polydopamine microspheres) or immunomodulators enable combinatorial therapy (Li et al., 2018; Fiedler et al., 2018; Ehlerding et al., 2018). 3) Advanced imaging: Microspheres co-loaded with ^99m^Tc or ^166^Ho allow SPECT/MRI-guided interventions (Son et al., 2021; Stella et al., 2020).

This review synthesizes the evolution of radioactive microspheres, from their physicochemical design to clinical applications, and highlights unresolved challenges in nuclear safety, imaging, and scalability. By critically evaluating recent preclinical and clinical data, we aim to outline a roadmap for next-generation theranostic microspheres in precision oncology.

## 2 Advances in the research of radioactive microspheres for the treatment of liver cancer

**FIGURE 1:**
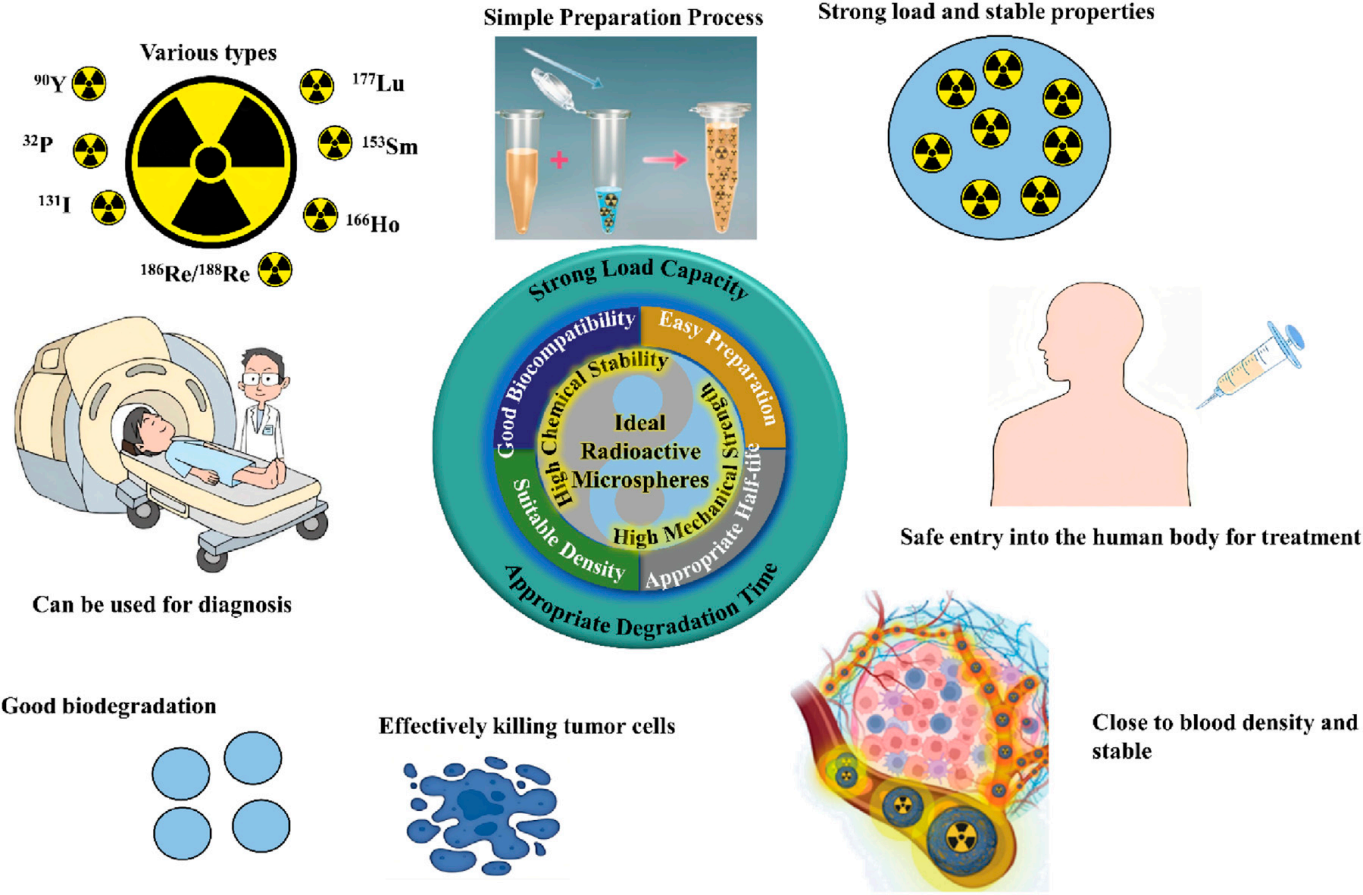
Properties of radioactive microspheres (Sun et al., 2025; Chen. et al., 2025).

Brachytherapy using radioactive microspheres has emerged as a promising approach for unresectable liver cancer and other solid tumors (Wang L. et al., 2025; Arranja et al., 2018; Nuzulia et al., 2024). Since the pioneering work with ^198^Au colloids, various radionuclides—including ^90^Y, ^32^P, ^131^I, ^188^Re, ^166^Ho, ^153^Sm, and ^177^Lu—have been developed to optimize therapeutic efficacy and diagnostic compatibility (Figure 1; Table 1). (Souza et al., 2022; Gulec et al., 2022; Abadie et al., 2021) Key challenges persist, such as radionuclide leakage, carrier biocompatibility, and precise tumor targeting (Pang et al., 2025). Recent advancements focus on multifunctional designs combining radiotherapy with imaging modalities and adjunct therapies (chemotherapy, photothermal) (Wu et al., 2023; Xiao et al., 2023; Bellendorf et al., 2024). This section comprehensively reviews the evolution, limitations, and innovations in radioactive microspheres, highlighting their clinical translation and future directions for hepatocellular carcinoma and other malignancies treatment.

**TABLE 1:** Basic properties of radioactive isotopes used for tumor treatment.

Radionuclide	Half-life (h)	Decay type	Tissue penetration (mm)	Average energy (MeV)	Maximum energy (MeV)	Imaging modality	Clinical status	Key advantages
^90^Y (Jiang et al., 2024; Pang et al., 2025)	64.1	β^-^	12	0.93	2.28	PET	FDA approved	Pure β^−^ emitter, high energy
^32^P (Alkatheeri et al., 2025)	343.2	β^-^	8	0.69	1.71	-	FDA approved	Pure β^−^ emitter, long half-life
^131^I (Cozmin et al., 2025)	193	β^−^、γ	2	0.19	0.61	SPECT	FDA approved	Well-established, dual capability
^186^Re (Cui et al., 2025)	90.6	β^−^、γ	4.5	0.35	1.07	SPECT	Clinical trials	Theranostic potential
^188^Re (Pang et al., 2025)	17.0	β^−^、γ	11	0.76	2.12	SPECT	Clinical trials	High energy, generator-produced
^166^Ho (Pang et al., 2025; Dos Reis et al., 2025)	26.8	β^−^、γ	8.7	0.67	1.85	SPECT	Clinical trials	MRI visibility (paramagnetic)
^153^Sm (Pang et al., 2025)	46.3	β^−^、γ	3	0.23	0.81	SPECT	FDA approved	Dual therapy/imaging
^99m^Tc (Wang et al., 2025b)	6.0	γ	-	-	0.14	SPECT	FDA approved	Ideal diagnostic imaging
^177^Lu (Zhang et al., 2025)	160.8	β^−^、γ	2.2	0.13	0.50	SPECT	FDA approved	Excellent imaging capability

### 2.1 ^90^Y microsphere

Yttrium-90 (^90^Y) is a beta-emitting radioisotope used for brachytherapy in liver cancer treatment due to its 12 mm tissue penetration range (Dong et al., 2024; Christie et al., 2011; Talebi and Rajabi, 2022). Early ^90^Y resin microspheres faced discontinuation due to significant ^90^Y leaching and adverse effects. Subsequent efforts focused on mitigating leaching by incorporating stable ^90^Y into Yttrium oxide (Y_2_O_3_) combined with alumina and silica, followed by high-temperature melting and neutron bombardment to create ^90^Y glass microspheres. This process reduced toxicity associated with ^90^Y leakage.

The Affiliated Zhongshan Hospital of Fudan University developed ^90^Y glass microspheres (30–50 μm) with a density of 3.27 g/mL, which require further clinical validation (Yan et al., 1993). High density can hinder injection, and limited microsphere count may inadequately irradiate large tumors. Resin microspheres, with their lower density, offer easier injection. To address ^90^Y leaching from resin microspheres, an alkali precipitation step was added post-ion exchange (Lau et al., 1994). This reduced the leakage rate while maintaining safety, enabling its usage for tumors with large blood supply. However, some leaching persisted, necessitating glucose or contrast agents for dispersion (Westcott et al., 2016). Metal ions in tumor tissue contributed to leakage rates of 1/1000 to 4/1000. Amthauer et al. detected radioactivity in urine from patients treated with ^90^Y resin microspheres, potentially from reversible ion exchange (Grosser et al., 2018). Commercial options include SIR-Spheres^®^ (polymer) and TheraSphere^®^ (glass), containing 1.2 million spheres per vial at 3 GBq activity (2500 GBq/microsphere). Glass microspheres are denser than blood and resin microspheres three times as much as blood (Baino et al., 2021). Zhen et al.'s pooled analysis of 16 studies demonstrates that TARE with ^90^Y microspheres achieves median overall survival of 14.3 months (95% CI: 11.9-17.1) in unresectable ICC (Intrahepatic Cholangiocarcinoma), with disease control rates of 77.2% (RECIST criteria: 11.5% partial response, 61.5% stable disease). Both glass (14.0 months OS) and resin (14.3 months OS) microspheres show comparable efficacy, with predominantly mild adverse events requiring no intervention (Zhen et al., 2019).

### 2.2 ^32^P microspheres

Phosphorus-32 (^32^P) is a pure beta-emitting radionuclide characterized by a half-life of 14.3 days and a maximum tissue penetration radius of 8 mm (Order et al., 1996; Rahman et al., 2020). These properties render ^32^P particularly suitable for long-distance transport and prolonged internal radiation therapy. Wallner et al. were the first to report the application of ^32^P colloidal chromic phosphate bound to albumin particles for brachytherapy of unresectable pancreatic tumors. However, the small particle size of ^32^P colloidal chromic phosphate and the high interstitial pressure of tumors may cause radioactive toxicity side effects when injected, no subsequent clinical reports using this method were seen. Masakazu Kawashita et al. from Kyoto University mixed Y_2_O_3_, Al_2_O_3_, and SiO_2_ and melted them at 1600°C to produce rectangular glass pieces material with a size of 1 cm in length and width, then bombarded red phosphorus with an electron beam to produce phosphorus ions (P^+^) and implanted them into the glass material to produce P^+^ rich glass material, which was finally bombarded by a high-flux neutron reactor to obtain ^32^P/^90^Y/^88^Y/^91^Y/^154^Eu glass microspheres (Kawashita et al., 1999). Therefore, Masakazu Kawashita from Kyoto University also successfully prepared YPO_4_ and Y_2_O_3_ microspheres with diameters ranging from 20 to 30 μm using a high-frequency induction heating plasma method (Kawashita, 2002). How to stably bind ^32^P onto microsphere carriers is an urgent problem that needs solving before applying ^32^P microspheres in clinical liver cancer brachytherapy treatments. Recent clinical studies demonstrate promising outcomes for ^32^P in pancreatic cancer treatment. A propensity-score weighted analysis of 104 LAPC patients showed that combining chemotherapy with EUS-guided ^32^P implantation significantly improved outcomes *versus* standard therapy (chemotherapy ± chemoradiotherapy). The combination therapy group exhibited 189 days longer restricted mean survival time (527.2 vs. 338.0 days), 168.6 days longer local progression-free survival, and 23.9% higher downstaging probability within 30 months. These results highlight ^32^P’s potential when combined with systemic therapy for localized tumor control (Lim et al., 2025).

### 2.3 ^131^I microsphere

Iodine-131 (^131^I) is a diagnostic and therapeutic radionuclide (half-life: 8.04 days) that emits both β-rays (99%) and γ-rays (1%). SPECT imaging can be performed, and its maximum tissue range radius is 2 mm (Larson et al., 2015; Li Y. et al., 2025). In 1992, Li et al. labeled gelatin microspheres with ^131^I and combined them with chemotherapeutic drugs to treat nine patients with unresectable liver cancer (Chen et al., 1992). However, the microspheres caused severe ectopic embolism, leading to one fatality due to systemic ^131^I leakage. Chen et al. synthesized biodegradable ^131^I/^125^I dual-labeled gelatin microspheres for rabbit liver embolization and metabolism studies using the chloramine T labeling method, and found that a small amount of ^131^I/^125^I would be released into the blood along with the degradation of gelatin microspheres as well as taken up by the thyroid gland (Ma et al., 2012). In 2016, Li et al. prepared ^131^I-gelatin-chitosan microspheres by reverse emulsion crosslinking method and chloramine T labeling method to overcome the problem of glass microspheres being non-degradable and unable to repeat brachytherapy treatment (Cai et al., 2016). Four years later, Li et al. synthesized a novel biodegradable chitosan-collagen composite microsphere labeled with ideal sedimentation efficiency, good *in vitro* and *in vivo* stability (Pang et al., 2020). Zhang et al. prepared a kind of silk protein microsphere labeled with ^131^I (^131^I-SFMs) for brachytherapy treatment of rat liver tumors by emulsification crosslinking method combined with chloramine T labeling method (Wu M. R. et al., 2022). Song et al. developed a type of poly (lactic acid) microsphere coated with copper sulfide nanoparticles and paclitaxel labeled with ^131^I (^131^I-HCuSNPs-MS-PTX), which can be used for SPECT and photoacoustic dual-modal imaging, as well as effective chemotherapy, radioembolization therapy, and photothermal therapy (Liu et al., 2018). Clinical studies of ^131^I SPECT/CT in differentiated thyroid cancer (DTC) demonstrate its prognostic value for lymph node metastases (LNM). A retrospective analysis of 942 DTC patients revealed that those without LNM achieved complete response (CR) faster (median 9.4 months vs. 44 months with LNM, HR 2.2) and had better progression-free survival (PFS, HR 0.46). Patients with enlarged ^131^I-negative LNM showed the longest time to CR (24 months), while those with small LNM had PFS comparable to LNM-negative cases. Treatment strategies should be individualized, with reoperation preferred for enlarged LNM (13.5 months to CR) and second radioiodine therapy for small LNM (better PFS, HR 4.0) (Heinrich et al., 2025).

Recent advancements in ^131^I microspheres focus on multifunctional designs. Sun et al. developed dual-functional ^131^I-PDA@PVA microspheres combining radioembolization and photothermal therapy (Sun et al., 2025). These microspheres exhibited high stability (76.5% retention in serum), excellent elasticity, and synergistic tumor inhibition (10% cell viability) via β/γ radiation and NIR-triggered hyperthermia (ΔT > 20°C). SPECT/CT enabled real-time tracking, demonstrating clinical potential for HCC theranostics.

### 2.4 ^186^Re/^188^Re microsphere

Rhenium-186 (^186^Re) and Rhenium-188 (^188^Re) (half-lives: 3.8 days and 0.71 days, respectively) are β- and γ-emitting radionuclides suitable for SPECT imaging with tissue range radii are 4.5 mm and 11 mm (Häfeli et al., 2007; Pourhabib et al., 2019). In 1998, Ehrhardt et al. prepared glass microspheres enriched with ^186^Re and ^188^Re by mixed ReO_2_ powder with glass frits and calcined them at 1050°C, then bombarded them with a neutron reactor (Conzone et al., 1998). However, the *in vitro* stability was poor. Hafeli et al. prepared a biodegradable ^186^Re/^188^Re polylactic acid microsphere by neutron reactor bombardment method (Häfeli et al., 1999; Häfeli et al., 2001). Subsequently, Hafeli et al. prepared ^186^Re/^188^Re fibrin glue with high adhesion strength in moist tissue by bombarding Re-enriched fibrinogen with a low-flux neutron reactor for intratumoral irradiation therapy (Häfeli et al., 2007). Mostafa et al. prepared a polylactic acid microsphere loaded with ^188^Re sulfide colloid nanoparticles, with a microsphere diameter range of 13–48 μm and a^188^Re labeling efficiency of up to 99% (Jamre et al., 2018). Lee et al. prepared a type of ^188^Re microsphere by mixing ^188^Re (OH_2_)_3_ (CO)^3+^, which was formed by the interaction of amino borane reduction of ^188^ReO_4_ and carbon monoxide, with human serum albumin microspheres, with a diameter of 13–48 μm, for the treatment of *in situ* hepatocellular carcinoma in rats (Ni et al., 2015), Saatchi et al. mixed polylactic acid containing dimethylpyridine amine with polycaprolactone as the dispersed phase and used microfluidic technology to prepare a type of mixed biodegradable microsphere for chelating labeling of ^188^Re (De La Vega et al., 2019). A Phase 1 trial (NCT01906385) evaluated ^186^Re-nanoliposomes (^186^RNL) delivered via convection-enhanced delivery in 21 recurrent glioblastoma patients. The maximum tolerated dose was not reached (up to 22.3 mCi), with most adverse events unrelated to treatment. Median overall survival was 11 months (17 months for patients receiving >100 Gy tumor absorbed dose vs. 6 months for <100 Gy), exceeding standard care outcomes. Disease control was achieved in 61.9% of patients (57.1% stable disease, 4.8% partial response), demonstrating ^186^Re’s potential for targeted brain tumor therapy (Brenner et al., 2025).

### 2.5 ^166^Ho microsphere

Holmium-166 (^166^Ho) possesses a half-life of 1.1 days, a maximum tissue range of 8.7 mm, and emits both β-rays and γ-rays (81 keV, 62%), making it a diagnostic-therapeutic nuclide for SPECT and magnetic resonance dual-modal imaging (Kühnel et al., 2022; Seevinck et al., 2012; Vente et al., 2014). Nijsen et al. prepared polylactic acid microspheres by solvent evaporation method, then added non-radioactive acetylacetone complexed ^165^Ho compound (^165^Ho-acetylacetone) to bind with polylactic acid microspheres (Nijsen et al., 1999; Nijsen et al., 2001). They filtered out microspheres with a particle size range of 20-50 μm through a filter screen and finally obtained ^166^Ho-acetylacetone-polylactic acid microspheres by bombarding them with a neutron reactor for 6 h. Under Good Manufacturing Practice (GMP) guidelines, Nijsen et al. optimized the evaporation temperature, sieving, and raw material selection in the solvent evaporation process to achieve gram-scale microsphere production. (Zielhuis et al., 2006). To improve the specific activity and ^166^Ho stability of ^166^Ho microspheres *in vivo*, Nijsen et al. formed two kinds of inorganic-like ^166^Ho microspheres (^166^HoPO_4_ microspheres and ^166^Ho(OH)_3_ microspheres) by ion exchange and neutron reactor irradiation of solid acetylacetone holmium microspheres with NaH2PO4 or NaOH (Arranja et al., 2020). A prospective phase 2 study (NCT03379844) in 31 HCC patients demonstrated the clinical utility of ^166^Ho-microspheres radioembolization. Hepatobiliary scintigraphy revealed significant reductions in treated liver volume (17%, p = 0.0027) and function (16%, p = 0.0017), with cirrhotic patients showing limited functional compensation (10% decrease vs. 0% in non-cirrhotics). The technique effectively maintained liver function in Child Pugh ≤ B7 patients (median MELD 9), with hepatic clearance rates correlating with biochemical markers (bilirubin, albumin, ALT; p < 0.05), supporting its safety profile in selected HCC populations (Reinders et al., 2025).

### 2.6 ^153^Sm microsphere

Samarium-153 (^153^Sm) is a radioactive nuclide that can release both β-rays with a maximum energy of 810 keV and γ-rays (103 keV), making it suitable for clinical SPECT imaging and radiotherapy (Wang et al., 2020; Bayouth et al., 1994; Wong et al., 2023). Bai et al. prepared a novel ^153^Sm-labeled biodegradable polylactide microsphere and studied its stability in rabbits. Yeong et al. labeled ^152^Sm on negatively charged acrylic microspheres by electrostatic adsorption then treated them with sodium carbonate solution for alkaline precipitation, and finally irradiated them with a neutron reactor for 6 h to obtain ^153^Sm microspheres (Wong et al., 2019). Subsequently, Yeong et al. used a solid-phase-oil-water solvent evaporation method to prepare ^152^Sm_2_O_3_ microspheres, which were then irradiated with a neutron reactor to obtain ^153^Sm microspheres with an average particle size of 33 μm, and no longer-lived radioactive nuclides or elemental impurities were found (Tan et al., 2022). Ying et al. also developed a biodegradable ^152^Sm-acetylacetone-polylactide microsphere for intra-arterial radioembolization therapy of liver tumors (Wong et al., 2020). Recent advances in ^153^Sm microspheres demonstrate their potential for combined chemo-radioembolization. Researchers successfully developed sulphonated polystyrene microspheres (31.95 ± 0.26 μm) co-loaded with [^153^Sm]Sm_2_O_3_ (2.82 ± 0.6 GBq/g) and doxorubicin (55.6% ± 1.1% encapsulation efficiency). These theranostic microspheres showed excellent radionuclide retention (>99% over 300 h) in physiological conditions with no detectable impurities after neutron activation. The formulation meets key requirements for intraarterial liver cancer treatment, offering simultaneous chemotherapy and radiotherapy delivery while maintaining optimal physicochemical properties for clinical translation (Wong et al., 2025).

### 2.7 Other radioactive microspheres

Ytterbium-175 (^175^Yb) is a radioactive isotope for studying *in vivo* biodistribution. In addition, Lutetium-177 (^177^Lu) is the metal nuclide for clinical diagnosis and treatment of diseases (O'Neill et al., 2020; Jamre et al., 2019). Shamsaei et al. designed and developed a novel biodegradable ^175^Yb-labeled polylactide microsphere for intratumoral irradiation radiotherapy embolization (Jamre et al., 2019). Gao et al. prepared a^177^Lu silica microsphere that can be used for SPECT imaging by directly physically mixing ^177^LuCl_3_ solution with mesoporous silica microspheres and then performing alkali precipitation, and directly used it for intratumoral injection therapy of tumor-bearing mice and achieved good anti-tumor efficacy (Wu X. et al., 2022).

Recent advancements in ^177^Lu-based therapies include the development of injectable 3D hollow porous granular hydrogels (^177^Lu-3D-HPGH) for precise brachytherapy (Xu et al., 2023). Synthesized via microfluidics and UV cross-linking, these hydrogels, developed by Xu et al., demonstrate high radiolabeling efficiency (97.85%), uniform tumor distribution, and robust anti-tumor efficacy in preclinical models, offering a promising theranostic platform for HCC treatment. Zhao et al. engineered ^177^Lu-PCMs using radiation-induced graft polymerization (Zhao et al., 2024). These phosphocholine-modified microspheres demonstrated ultra-stable Lu coordination (DFT-confirmed chelation), mechanical robustness (117.2 μm size), and precise tumor targeting in rabbit VX2 models. SPECT/CT-guided intra-arterial brachytherapy achieved complete tumor regression without ectopic leakage, highlighting translational potential for image-guided HCC treatment.

Innovative radio-immunotherapy approaches have emerged. Yang et al. created ^177^Lu-labeled alginate microspheres co-loaded with IDO1 inhibitor Indoximod (Yang et al., 2023). The 2 μm microspheres achieved >90% labeling efficiency, suppressed kynurenine pathways, and enhanced CD8^+^ T-cell infiltration. Combined with αPD-L1, they inhibited distal tumors in H22 models via DC maturation and Treg downregulation, showcasing a promising immunomodulatory platform.

In summary, most traditional radioactive microspheres are prepared by “cold” microspheres using methods such as melt spraying, solvent evaporation, inverse emulsion cross-linking, and alkali precipitation. Finally, they all need to be bombarded by high-flux neutron reactors to activate the “cold” microspheres into radioactive microspheres.

## 3 Future prospect of clinical application of radioactive microspheres

Compared with conventional therapies, radioactive microspheres offer distinct advantages and limitations. Versus TACE, microspheres provide more sustained radiation exposure (weeks vs. days) with better tumor penetration but require specialized nuclear facilities (Nuzulia et al., 2024; Welling et al., 2023). Relative to systemic therapies (sorafenib, lenvatinib), they demonstrate higher local control rates with fewer systemic side effects, though lack distant disease control (Nuzulia et al., 2024).

^90^Y microspheres are representative example of radioactive microspheres to analyze a series of clinical problems. The range of TACE and brachytherapy will no longer be limited to HCC with the development of technology. For instance, in bone cancer treatment, radionuclide-doped hydroxyapatite microspheres serve as bone graft scaffolds, where beta emitters deliver localized high-dose radiation to kill cancer cells. And hydroxyapatite microspheres can also promote bone tissue growth and regeneration as scaffolds (Nuzulia et al., 2024). Whether it is innovation based on material or radioactive elements, the area of application and the route of treatment will be greatly developed in the future. By the delivery of efficient drug and targeted delivery, a lot of non-radioactive microspheres have been combined many immunotherapies, such as boron neutron capture therapy (BNCT) and photodynamic therapy (PDT).

Emerging technologies like SHIFT (Superstable Homogeneous Iodinated Formulation) are reshaping brachytherapy. Chen et al. developed radiolipiodol via CO_2_ supercritical fluid, achieving ≥99% labeling efficiency and > 2-week tumor retention in preclinical models (Chen et al., 2025). Clinical trials confirmed minimal ^18^F leakage (T/N ratio ≥50), offering a paradigm shift in stable nuclide encapsulation for precision HCC therapy. Regulatory pathways for novel agents (e.g., ^177^Lu) face challenges: FDA requires standardized dosimetry protocols, while EMA mandates comparative efficacy data. Centralized ^177^Lu production could reduce costs *versus* decentralized ^90^Y systems. Practical adoption hinges on streamlining supply chains and establishing clear criteria for combination therapies (Pang et al., 2025).

Looking forward, radioactive microspheres can potentially treat cancers efficiently through three key research directions: 1) Personalized dosimetry optimization using AI-based tumor perfusion analysis to predict microsphere distribution patterns (Dong et al., 2025); 2) Development of “smart” microspheres with stimuli-responsive drug release (pH, enzyme, or temperature-activated) for precision combination therapies (Li J. et al., 2025); and 3) Standardization of radio-immunotherapy protocols combining PD-1/PD-L1 inhibitors with radionuclides to enhance abscopal effects (Li et al., 2024). Critical technological gaps remain in real-time intraprocedural dosimetry systems and scalable manufacturing of multifunctional microspheres. The integration of theranostic radionuclides with advanced biomaterials may enable truly personalized treatment regimens based on tumor molecular profiling. These innovations could revolutionize locoregional cancer therapy while maintaining manageable toxicity profiles.

## 4 Conclusion

Radioactive microspheres have emerged as a pivotal modality in the locoregional treatment of non-surgically resectable solid tumors, particularly in hepatocellular carcinoma and other malignancies. This review delineates the evolution, challenges, and future directions of these innovative therapeutic agents. The principle of delivering high-dose radiation directly to tumor vasculature allows for effective tumor ablation while preserving surrounding healthy tissues, a core aspect of brachytherapy. Key advancements in material science and radiochemistry have enabled the design of multifunctional microspheres that combine therapeutic and imaging capabilities. For instance, radionuclides such as ^90^Y and ^166^Ho have shown clinical promise due to their optimal tissue penetration and compatibility with imaging modalities like SPECT. Nevertheless, challenges such as radionuclide leakage, imaging limitations, and dose heterogeneity remain, necessitating enhanced predictive dosimetry models and improved material formulations. Recent innovations include engineered glass microspheres that mitigate leakage and designs integrating targeted drug delivery systems, such as radio-immunotherapy approaches. As technological advancements continue, the landscape of radioactive microspheres is set to expand significantly, fostering new applications beyond traditional cancer therapies and enhancing the precision of locoregional management strategies. Thus, ongoing research into optimizing these therapeutic platforms is crucial for advancing the field of precision oncology.
