# Correction: Advancements in the investigation of radioactive microspheres for brachytherapy

**DOI:** 10.3389/fbioe.2025.1715928

**Published:** 2026-02-27

**Authors:** Xiaohui Jiang, Lei Chen, Xiao Xu

**Affiliations:** 1 College of Physics and Electronic Engineering, Jining University, Jining, China; 2 Cancer Center, Dongguan Engineering Research Center for Innovative Boron Drugs and Novel Radioimmune Drugs, The 10th Affiliated Hospital of Southern Medical University, Southern Medical University, Guangzhou, China; 3 Guangdong Engineering Research Center of Boron Neutron Therapy and Application in Malignant Tumors, The 10th Affiliated Hospital of Southern Medical University, Southern Medical University, Guangzhou, China

**Keywords:** tumor, radiation, brachytherapy, radioactive microspheres, theranostics

**Affiliation** “College of Physics and Electronic Engineering, Jining University, Jining, China” was erroneously omitted for **Author** Xiaohui Jiang. This **Affiliation** has now been added for author Xiaohui Jiang.

**Author** Xiaohui Jiang was erroneously assigned to **Affiliations** “1Cancer Center, Dongguan Engineering Research Center for Innovative Boron Drugs and Novel Radioimmune Drugs, The 10th Affiliated Hospital of Southern Medical University, Southern Medical University, Guangzhou, China” and “2Guangdong Engineering Research Center of Boron Neutron Therapy and Application in Malignant Tumors, The 10th Affiliated Hospital of Southern Medical University, Southern Medical University, Guangzhou, China”. These **Affiliations** have now been removed for **Author** Xiaohui Jiang.

The original article has been updated.

